# Saliva for assessing creatinine, uric acid, and potassium in nephropathic patients

**DOI:** 10.1186/s12882-019-1437-4

**Published:** 2019-07-04

**Authors:** Giancarlo Bilancio, Pierpaolo Cavallo, Cinzia Lombardi, Ermanno Guarino, Vincenzo Cozza, Francesco Giordano, Giuseppe Palladino, Massimo Cirillo

**Affiliations:** 1 Department “Scuola Medica Salernitana”, University of Salerno, Baronissi, SA Italy; 2 Nephrology Clinic, University Hospital, Salerno, SA Italy; 3 Department of Physics, University of Salerno, Fisciano, SA Italy; 4 Rummo Hospital, Benevento, BN Italy; 5 Department Public Health, University of Naples “Federico II”, Via Sergio Pansini, 5, 80131 Naples, Italy

**Keywords:** Saliva, Plasma, Creatinine, Uric acid, Potassium, Kidney

## Abstract

**Background:**

Lab tests on saliva could be useful because of low invasivity. Previous reports indicated that creatinine, uric acid, and potassium are measurable in saliva. For these analytes the study investigated methodology of saliva tests and correlations between plasma and saliva levels.

**Methods:**

The study enrolled 15 healthy volunteers for methodological analyses and 42 nephropathic patients for plasma-saliva correlations (35 non-dialysis and 7 dialysis). Saliva was collected by synthetic swap right after venipuncture for blood withdrawal. Blood and saliva, unless otherwise indicated, were collected early in the morning after overnight fast and lab tests were performed in fresh samples by automated biochemistry (standard). Methodological analyses included blind duplicates, different collection mouth sites, day-to-day variability, different collection times, and freezing-thawing effects. Analyses on plasma-saliva correlations included post-dialysis changes.

**Results:**

For saliva lab tests of all analytes, blind duplicates, samples from different mouth sites or of different days were not significantly different but were significantly correlated (differences ≤14.4%; R ≥ 0.620, *P* ≤ 0.01). For all analytes, mid-morning saliva had lower levels than but correlated with standard saliva (differences ≥15.8%; R ≥ 0.728, P ≤ 0.01). Frozen-thawed saliva had lower levels than fresh saliva for uric acid only (− 17.2%, *P* < 0.001). Frozen-thawed saliva correlated with fresh saliva for all analytes (R ≥ 0.818, *P* ≤ 0.001). Saliva and plasma levels differed but correlated with plasma for creatinine (R = 0.874, *P* < 0.001), uric acid (R = 0.821, P < 0.001) and potassium (R = 0.767, P < 0.001). Post-dialysis changes in saliva paralleled post-dialysis changes in plasma.

**Conclusion:**

Saliva levels of creatinine, uric acid, and potassium are measurable and correlated with their plasma levels. Early morning fasting fresh saliva samples are advisable because later collection times or freezing lower the saliva levels of these analytes.

## Introduction

The use of saliva for lab tests has been investigated in various medical areas because of the non-invasivity of saliva collection [1–4]. Saliva lab tests could be acceptable for practical scopes if they were of use in detection and/or follow-up of certain diseases or if their results paralleled the results of standard tests in other biological samples. Previous observations indicated that correlations between plasma concentrations and saliva concentrations are detectable for some but not all analytes [1–4]. Plasma levels of creatinine, uric acid, and potassium are important for diagnosis and follow-up of several disorders including kidney disease and gout. There is evidence that the three analytes are measurable in saliva [5–14]. Potassium is the most abundant salivary cation and its concentration is controlled by cellular transporters of the salivary duct [15]. The mechanisms accounting for the presence in saliva are undefined for creatinine and uric acid. The diffusion through cell membrane or intercellular junctions is considered difficult due to the low lipid solubility of creatinine and uric acid but could increase when plasma levels are high [3]. Theoretically, salivary glands could express transporters similar to those ones that in the epithelial cells of the renal tubule account for the renal excretion of creatinine and uric acid through tubular secretion [16, 17]. Blood contamination of saliva could be an additional possibility [18]. Research data are missing for methodological aspects as technical error in blind duplicates, effects of collection in different mouth sites, differences between times of collection, and effects of freezing/thawing. As part of a project on space medicine (https://www.nasa.gov/mission_pages/station/research/experiments/explorer/Investigation.html?#id=304), the present study was therefore designed to investigate on saliva measurements of creatinine, uric acid, and potassium with specific focus on methodological aspects and on associations between plasma levels and saliva levels.

## Methods

This is an observational study that was performed in accordance with the ethical principles of the Declaration of Helsinki and was approved by the local institutional Ethics Committees (n. 5/2012 and 4/2013). The study was included in a collaboration project developed by the Italian Space Agency (*Agenzia Spaziale Italiana*, ASI) and the National Aeronautics and Space Administration (NASA) for investigation on the use of saliva in the monitoring of metabolic changes during space mission (https://www.nasa.gov/mission_pages/station/research/experiments/explorer/Investigation.html?#id=304), [19, 20]. The study enrolled healthy volunteers of the department personnel for analyses on methodological aspects and patients with chronic kidney disease (CKD) of the outpatient clinic of the University Hospital for analyses on associations saliva/plasma as previously reported [19]. CKD patients were selected for analyses on associations saliva/plasma because kidney disease is known to induce sizeable increases in plasma concentrations of all analytes under study.

For healthy volunteers, exclusion criteria were acute disease, or chronic disease, or pharmacological treatment. For nephropathic patients, the selection criterion was the standard CKD diagnosis [21]. At least five CKD patients were enrolled per each CKD stage to have a kidney function range from normal to severely reduced. The enrollment included patients on chronic hemodialysis for assessment of the dialysis effects on saliva analytes. Smokers were excluded from both groups given the effects of this habit on saliva [22]. All participants signed an informed consent.

Blood samples were collected early in the morning after an overnight fast, a schedule that was in accordance with the standard work-up in the outpatient CKD clinic. In dialysis patients, the blood withdrawal was repeated at the end of the morning, 4-h, standard dialysis session in accordance with current guidelines [23]. Samples of saliva were collected always 1–2 min after blood. To comply with the restraints of space missions, saliva was collected with the use of a synthetic swap (Salivette, Sarstedt, Germany) and without timing or stimulation with paraffin or other agents [19]. Blood contamination was controlled by macroscopic inspection only [18]. Blood samples were rapidly centrifuged for plasma separation. The swap was rapidly centrifuged for saliva separation and mucin removal. Unless otherwise indicated, lab tests were performed using fresh samples of plasma and saliva (standard). Lab measurements were done by fully automated biochemistry with the use of commercially available kits for creatinine (enzymatic method), uric acid (uricase), and potassium (indirect ion selective potentiometry) (Abbott, Illinois, US) [24].

Analyses on methodological aspects included five objectives: differences between blind duplicates of standard samples; differences between standard samples simultaneously collected in two different sites (i.e., right vestibulum oris and left vestibulum oris); differences between standard samples of different days (day-to-day variability); differences between standard samples and mid-morning samples (i.e., samples collected 3-h after the completion of breakfast); differences between standard samples and frozen/thawed samples. The timing of mid-morning samples was selected to match the schedule of experiments in space mission [19]. In experiments on frozen/thawed saliva, standard samples were divided in two aliquots: one was maintained at 2–4 °C for one hour while the other one was frozen and thawed at 2–4 °C. Analyses on associations saliva/plasma for the three analytes investigated the associations between increases in plasma concentrations and increases in saliva concentrations.

Statistical procedures included Student’s T-test for paired observations, Pearson correlation, linear regression, and Bland-Altman plots [25]. The report of results included mean, SEM, Pearson correlation coefficient (R), and regression coefficient with 95% confidence intervals (95%CI).

## Results

### Descriptive statistics

Mean ± SEM of age was 35 ± 5 years in the 15 individuals of the control group (9 men and 6 women) and 36 ± 3 years in the 35 patients of the CKD group (19 men and 16 women). Table 1 reports clinical characteristics of the CKD group. All the 7 patients with CKD stage 5 were on hemodialysis treatment since at least eight months. Detection limits for lab tests were 9 μmol/L for creatinine, 60 μmol/L for uric acid, and 2 mmol/L for potassium. No saliva sample was with visible blood contamination. Saliva creatinine was below the detection limit in one control and eight CKD patients. Within the subgroup with non-measurable saliva creatinine, plasma creatinine was 68 μmol/L in the healthy control and ranged from 78 to 141 μmol/L in CKD patients. Saliva levels of uric acid and potassium were above the detection limit in all cases.

**Table 1:** Clinical characteristics of the group with chronic kidney disease (CKD)

CKD stage	Number of patients	Number of patients by diagnosis
1	5	nephrosclerosis (*n* = 1), diabetic nephropathy (*n* = 2), polycystic kidney disease (n = 2)
2	8	nephrosclerosis NS (*n* = 4), diabetic nephropathy (n = 2), polycystic kidney disease (n = 2)
3	10	nephrosclerosis (n = 2), diabetic nephropathy (*n* = 3), polycystic kidney disease (n = 3), glomerulonephritis (n = 2)
4	5	diabetic nephropathy (n = 2), polycystic kidney disease (n = 1), glomerulonephritis (n = 1), pyelonephritis (n = 1)
5	7	diabetic nephropathy (n = 2), polycystic kidney disease (n = 2), glomerulonephritis (n = 1), unknown (n = 2)

CKD stage was defined as per guidelines using estimated Glomerular Filtration Rate (eGFR) in the presence of pathologic abnormalities or markers of kidney damage [21]. eGFR thresholds for CKD stage were as follows (mL/min × 1.73 m^2^ of body surface area): stage 1 = eGFR equal to or greater than 90, stage 2 = eGFR 89–60, stage 3 = eGFR 59–30, stage 4 = eGFR 29–15, stage 5 = eGFR less than 15

### Methodological analyses in healthy controls

Table 2 summarizes the results for analyses in the control group on blind duplicates, differences between sites of collection, and day-to-day variability, time of collection, and freezing/thawing effects. Means saliva concentrations of creatinine, uric acid, and potassium differed by < 5% between duplicate samples and were highly correlated with each other. The values of the R between duplicates ranged > 0.99 for uric acid and potassium but < 0.75 for creatinine. Findings were similar in comparisons between simultaneous saliva samples from different sites of collection. In analyses on day-to-day variability, mean values differed by < 2.3% for creatinine and uric acid and by 14.4% for potassium. The values of the R between different days tended to be lower as compared to data in duplicates for all analytes. Compared to standard early morning saliva, mid-morning saliva had 15–30% lower concentrations of creatinine, uric acid, and potassium. The R values between concentrations in standard saliva and concentrations in mid-morning saliva were highly significant and of magnitude similar to that of duplicate samples. Saliva freezing/thawing did not affect the measurements of creatinine and potassium but reduced by 17.2% the level of measurable uric acid. The R values between fresh saliva and frozen/thawed saliva were similar to those between duplicates.

**Table 2:** Methodological analyses in saliva lab tests for creatinine, uric acid, and potassium in control group: blind duplicates, site of collection, day-to-day variability, time of collection, and freezing/thawing effect (mean ± SEM)

			*P*	*R*
Blind duplicates	*first duplicate*	*second duplicate*		
Creatinine, μmol/L	11.5 ± 3.9	11.7 ± 1.5	*0.321*	*0.748* ^*§*^
Uric acid, μmol/L	261 ± 166	251 ± 145	*0.187*	0.993*
Potassium, mmol/L	22.2 ± 6.6	22.5 ± 5.9	*0.349*	0.997*
Site of collection	*right vestibulum*	*left vestibulum*		
Creatinine, μmol/L	11.7 ± 4.1	11.4 ± 1.6	*0.628*	*0.728* ^*§*^
Uric acid, μmol/L	262 ± 74	264 ± 65	*0.865*	0.991*
Potassium, mmol/L	23.2 ± 7.5	23.6 ± 5.8	*0.791*	0.898*
Day-to-day variability	*first day*	*second day*		
Creatinine, μmol/L	11.5 ± 3.9	11.5 ± 2.0	*0.723*	*0.735* ^*§*^
Uric acid, μmol/L	261 ± 166	255 ± 165	*0.790*	*0.677* ^*§*^
Potassium, mmol/L	22.2 ± 6.6	25.4 ± 6.7	*0.389*	*0.620* ^*§*^
Time of collection	*standard sample*	*mid-morning sample*		
Creatinine, μmol/L	11.5 ± 3.9	9.1 ± 1.5	*0.038*	*0.765*
Uric acid, μmol/L	261 ± 166	184 ± 102	*< 0.001*	*0.915*
Potassium, mmol/L	22.2 ± 6.6	18.7 ± 5.9	*< 0.001*	*0.728*
Freezing/thawing effects	*standard sample*	*frozen/thawed sample*		
Creatinine, μmol/L	11.5 ± 3.9	10.5 ± 1.4	*0.488*	*0.899*
Uric acid, μmol/L	261 ± 166	216 ± 118	*< 0.001*	*0.818*
Potassium, mmol/L	22.2 ± 6.6	21.4 ± 5.7	*0.389*	*0.829*

Non-stimulated fresh saliva collected early in the morning after an overnight fast with the use of a synthetic swap

Control group *N* = 15 (14 for creatinine)

* *P* < 0.001, ^§^ *P* ≤ 0.01

Saliva and plasma samples significantly differed for creatinine concentration (mean ± SEM in saliva and plasma = 11.1 ± 0.3 and 84.2 ± 1.7 μmol/L, *P* < 0.001 by t-test for paired data), uric acid concentration (206 ± 48 and 291 ± 52 μmol/L, *P* = 0.037), and potassium concentration (25.2 ± 3.3 and 4.08 ± 0.2 mmol/L, *P* < 0.001).

### Association analyses in nephropathic patients

**Fig. 1:**
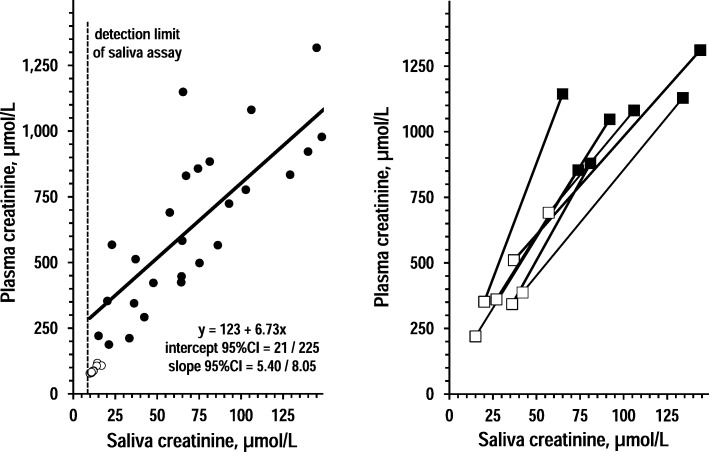
Association between saliva creatinine and plasma creatinine. Left panel: individual values and regression line of plasma creatinine over saliva creatinine in 14 controls and 27 CKD patients (open circles and closed circles, respectively). The insert reports equation and coefficients with 95%CI in CKD patients. The dotted grey line indicates the detection limit of the assay for saliva creatinine. Right panel: individual values of pre-dialysis values of plasma creatinine and saliva creatinine over post-dialysis values of plasma creatinine and saliva creatinine in 7 dialysis patients (pre-dialysis: closed squares; post-dialysis: open squares)

**Fig. 2:**
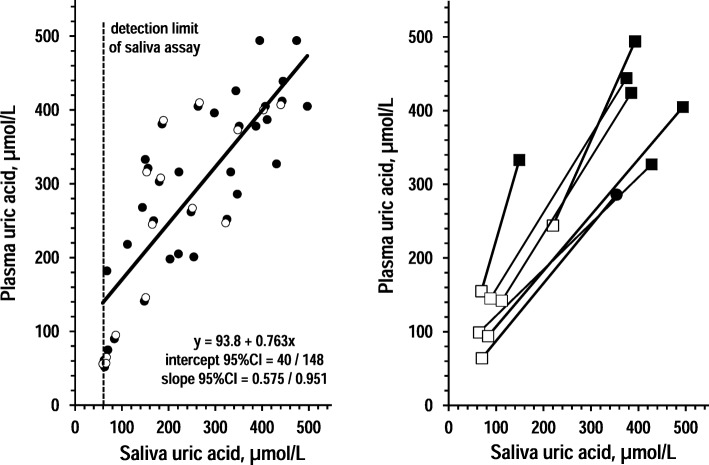
Association between saliva uric acid and plasma uric acid. Left panel: individual values and regression line of plasma uric acid over saliva uric acid in 15 controls and 35 CKD patients (open circles and closed circles, respectively). The insert reports equation and coefficients with 95%CI in CKD patients. The dotted grey line indicates the detection limit of the assay for saliva uric acid. Right panel: individual values of pre-dialysis values of plasma uric acid and saliva uric acid over post-dialysis values of plasma uric acid and saliva uric acid in 7 dialysis patients (pre-dialysis: closed squares; post-dialysis: open squares)

**Fig. 3:**
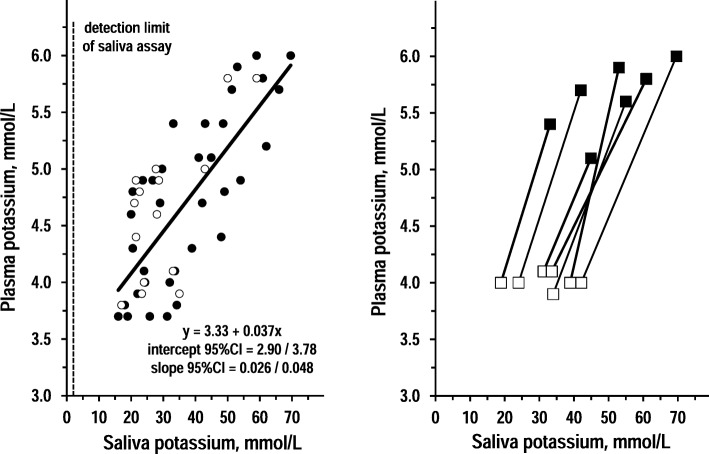
Association between saliva potassium and plasma potassium. Left panel: individual values and regression line of plasma potassium over saliva potassium in 15 controls and 35 CKD patients (open circles and closed circles, respectively). The insert reports equation and coefficients with 95%CI in CKD patients. The dotted grey line indicates the detection limit of the assay for saliva uric acid. Right panel: individual values of pre-dialysis values of plasma potassium and saliva potassium over post-dialysis values of plasma potassium and saliva potassium in 7 dialysis patients (pre-dialysis: closed squares; post-dialysis: open squares)

Saliva and plasma samples were significantly different for creatinine concentration (61 ± 8 and 533 ± 62 μmol/L, *P* < 0.001), uric acid concentration (258 ± 23 and 289 ± 21 μmol/L, *P* = 0.018), and potassium concentration (37.6 ± 2.6 and 4.73 ± 0.13 mmol/L, *P* < 0.001). Plasma concentrations and saliva concentrations were significantly correlated for creatinine (R = 0.874, P < 0.001), uric acid (R = 0.821, P < 0.001), and potassium (R = 0.767, P < 0.001). Figures 1, 2 and 3 show the associations of saliva concentrations with plasma concentrations for creatinine, uric acid, and potassium in controls and CKD patients. For all three analytes, participants with higher saliva concentrations had also higher plasma concentrations (left panels of Figs. 1, 2 and 3). In the subgroup of dialysis patients, an association was apparent for all analytes between post-dialysis decreases in saliva concentrations and post-dialysis decreases in plasma concentrations (right panels of Figs. 1, 2 and 3).

**Fig. 4:**
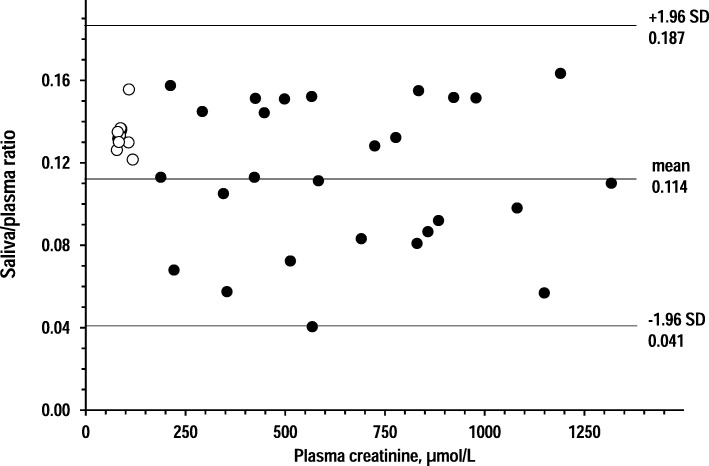
Bland-Altman plots of saliva/plasma ratio of creatinine over plasma creatinine in 14 controls and 27 CKD patients (open circles and closed circles, respectively). Horizontal lines are drawn at the mean difference, and at the limits of agreement, which are defined as the mean difference plus and minus 1.96 times the standard deviation of the differences in the CKD group

**Fig. 5:**
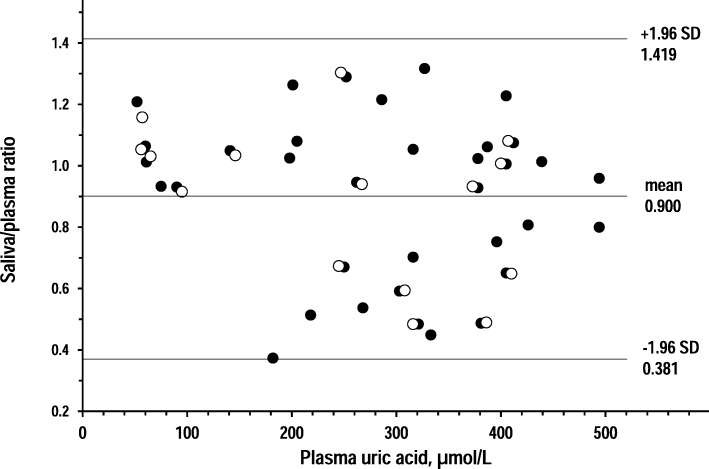
Bland-Altman plots of saliva/plasma ratio of uric acid over plasma uric acid in 15 controls and 35 CKD patients (open circles and closed circles, respectively). Horizontal lines are drawn at the mean difference, and at the limits of agreement, which are defined as the mean difference plus and minus 1.96 times the standard deviation of the differences in the CKD group

**Fig. 6:**
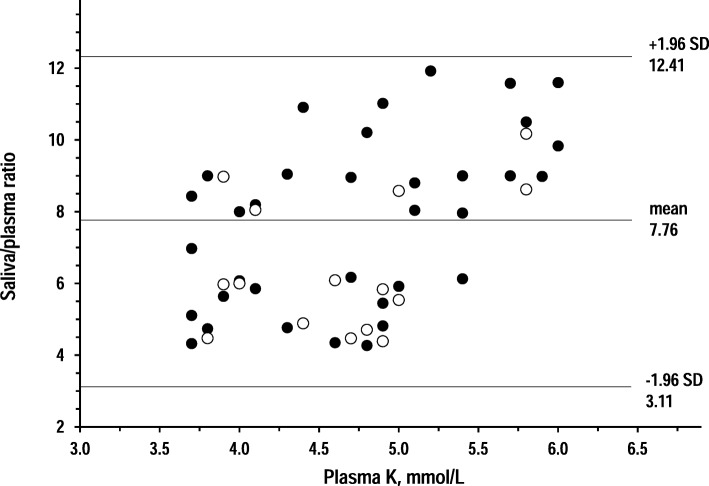
Bland-Altman plots of saliva/plasma ratio of potassium over plasma potassium in 15 controls and 35 CKD patients (open circles and closed circles, respectively). Horizontal lines are drawn at the mean difference, and at the limits of agreement, which are defined as the mean difference plus and minus 1.96 times the standard deviation of the differences in the CKD group

Figures 4, 5 and 6 show Bland-Altman plots that investigated whether the relationship between plasma tests and saliva tests varied along the range of plasma concentrations. For all three analytes, higher plasma concentrations related to larger deviations from plasma tests but not to trends toward more positive bias or more negative bias.

## Discussion

The present study investigated two separate objectives: in healthy controls, the methodological aspects of lab tests for measurements of creatinine, uric acid, and potassium in untimed samples of saliva; in CKD patients, the associations of the three analytes between plasma concentrations and saliva concentrations.

Main limitations of the study were the sample size, the lack of information for ethnic groups, for pediatric ages, for older ages, for other diseases, for peritoneal dialysis, for saliva flow rate, and for possible blood contamination of saliva. The present study, therefore, could not assess whether or not the control for the confounding of saliva flow or blood contamination of saliva affected the relationships between plasma tests and saliva tests. Theoretically, blood contamination could have opposite confounding effects depending on the ratio of the saliva/plasma concentration of the given analyte, that is an increase in the saliva concentration of analytes with saliva concentration much lower than plasma concentration as creatinine or, vice versa, a decrease in the saliva concentration of analytes with saliva concentration much higher than plasma concentration as potassium.

Data in healthy controls indicated that, for all three analytes, the technical error of saliva tests was < 5% and the correlation coefficients between coupled measurements were > 0.7 when evaluated either between blind duplicates of the same sample and between simultaneous saliva samples from different mouth sites. The accuracy of results could differ in the case of stimulated or timed saliva collections. Day-to-day intra-individual variability of saliva potassium was apparently higher in comparison to either the technical error of saliva potassium measurements and to the day-to-day variability of other analytes. This variability could reflect the influence of dietary factors although other possibilities could not be excluded. The concentrations of all three analytes were appreciably lower in mid-morning saliva collected three hours after the breakfast as compared to early morning saliva collected under fasting conditions. This datum agreed with the report of lower saliva osmolality in mid-morning samples compared to early morning samples [26] and pointed to circadian differences in saliva dilution although other mechanisms cannot be excluded. Frozen/thawed saliva had significantly lower concentrations of uric acid, not of creatinine and potassium, a difference which likely reflected the different solubility of the analytes. The lack of previous data on technical error, day-to-day variability, freezing/thawing effects precluded the comparison with other studies.

Saliva and plasma concentrations of the three analytes differed consistently either in controls and patients. Compared to plasma, early morning creatinine saliva concentration under fasting condition was several times lower and nevertheless measurable in most of participants with the use of automated biochemistry and a commercially available enzymatic kit. Compared to plasma, early morning saliva concentrations under fasting conditions were slightly lower for uric acid but several times higher for potassium. These findings agreed with the results of several other studies [5–14].

Data in CKD patients indicated that there were positive, linear relationships between saliva concentrations and plasma concentrations of creatinine, uric acid, and potassium. Present findings about the associations between plasma and saliva for creatinine and uric acid were consistent with the conclusions of several other reports [5–14]. The present finding of a positive association between plasma and saliva also for potassium actually disagreed with the conclusions of two previous studies that could not find such a positive association [8, 14]. The lack of a positive association for saliva potassium in those two studies likely reflected the confounding effect of circadian rhythms because saliva was collected in those studies at different times of the day after a 1–2 h fast.

Regardless of the mechanisms accounting for their presence in saliva, the evidence of associations saliva/plasma pointed to the possibility that the plasma level of creatinine, uric acid, and potassium plays a key-role in the regulation of secretion and/or diffusion of these analytes from plasma into saliva. The observations of parallel post-dialysis changes of the three analytes in plasma and in saliva indicated that the saliva/plasma associations reflected rapid interchanges between plasma and saliva.

The practical implications of the study concerned the possible uses of saliva for screening, monitoring, or follow-up of kidney dysfunction and associate disorders. All over, the non-invasivity of saliva sampling makes saliva suitable when the collection of blood samples would be difficult or undesirable as in the case of children, arduous venipuncture, need of many repeated samples, peritoneal dialysis, etc. The present set of data proved that reliable saliva lab tests are feasible even using saliva samples collected without stimulation or timing. The evidence of differences between early morning samples and mid-morning samples indicated the need of standardized conditions to reduce the confounding of circadian rhythms. Moreover, data about the freezing/thawing effects indicated a significant bias of these procedures for the assessment of saliva uric acid. Considering these methodological restraints and the large differences in absolute concentrations of the analytes between saliva and plasma, it is possible to propose that changes in saliva concentrations of creatinine, uric acid and potassium could be used as indirect indices of parallel, proportional changes in their plasma concentrations.

Briefly, the study reported new observations about the measurements in saliva of creatinine, uric acid, and potassium and about the associations between saliva and plasma for these analytes. The results indicated the saliva tests were feasible by commercially available automated biochemistry, were accurate and correlated with blood tests. Study results indicated that the time of the saliva collection and the freezing/thawing procedure affect the lab tests on saliva.

## Data Availability

The datasets used and/or analyzed during the current study are available from the corresponding author on reasonable request.
